# Neutrophil-to-lymphocyte ratio as a predictor of short- and long-term complications in pediatric burns

**DOI:** 10.1186/s13052-024-01834-3

**Published:** 2025-01-23

**Authors:** Carlos Delgado-Miguel, Lara Fuentes Gómez, Ada García Morán, Miriam Miguel-Ferrero, Mercedes Díaz, Juan Carlos López-Gutiérrez

**Affiliations:** 1 Department of Pediatric Surgery, Fundación Jiménez Díaz University Hospital, Avenida de los Reyes Católicos, 2, Madrid, 28040 Spain; 2 Institute for Health Research IdiPAZ, La Paz University Hospital, Madrid, Spain; 3 Department of Pediatric Surgery, La Paz Children´s University Hospital, Madrid, Spain

**Keywords:** Neutrophil-to-lymphocyte ratio, Pediatric burns, Sequelae, Predictive factor

## Abstract

**Background:**

Neutrophil-to-Lymphocyte Ratio (NLR) has been postulated as a useful inflammatory biomarker in the prediction of complications in different pediatric diseases. Our aim is to analyze the predictive value of NLR in the development of complications in burned children, both in the short-term (need for grafting) and in the long-term (need for surgery of the sequelae).

**Methods:**

A retrospective study was performed on burned patients under 18-years admitted to our Burn Unit between 2015 and 2021. Demographic, clinical and laboratory data at admission were evaluated. Predictive factors for the development of complications after burns (time of evolution, burned total body surface area, and acute phase reactants) were analyzed using sensitivity and specificity analysis (ROC curves).

**Results:**

A total of 342 patients (198 males, 144 females) were included, with a median age of 27 months (interquartile range 15–83 months). In 97.4% of the cases, burns were primarily caused by thermal injuries (78.4% scald burns). Acute escharectomy and grafting were performed in 85 patients (24.9%), while long-term sequelae were observed in 112 cases (32.7%). NLR was the most sensitive and specific predictor for the need for escharectomy and grafting (Sensitivity 90%, Specificity 88.4%; AUC 0.920), for the development of long-term sequelae (Sensitivity 80.4%, Specificity 83.5%; AUC 0.849) and for the need for surgery of the sequelae (Sensitivity 83.5%, Specificity 80.9%; AUC 0.833).

**Conclusion:**

NLR may be considered a useful predictor for the development of short- and long-term complications in childhood burns. It may help in the identification of high-risk patients to prevent sequelae.

## Background

Pediatric burns remain a major public health problem due to their mortality, long-term sequelae and associated costs [1, 2]. Several predictors of post-burn mortality and related complications have been described, such as body surface area burned, depth of burn, need for invasive ventilation and admission to the Intensive Care Unit [3, 4]. Identification of predictive factors for a worse outcome in these patients is essential to prevent unfavorable consequences.

Inflammation in burn-injured patients is the main pathophysiological mechanism that causes morbidity and mortality [5]. Recently, the Neutrophil-to Lymphocyte Ratio (NLR) has been postulated as an inflammatory biomarker in different pediatric diseases associated with a high degree of inflammatory response, such as Henoch-Schönlein purpura, intussusception or acute appendicitis [6, 7]. Its usefulness has been described both in the diagnosis and prognosis of these diseases, as well as a good predictor of the development of complications [8, 9].

Burn injuries involve a local inflammatory lesion that triggers a systemic inflammatory response. In this context, NLR has been identified as a prognostic factor for acute kidney injury and in-hospital mortality in adult patients with extensive burns [10, 11]. However, the relationship between inflammatory response and burn morbidity has not been analyzed to date. The aim of this study is to analyze the role of the Neutrophil-Lymphocyte ratio as a morbidity predictor in terms of short-term complications and long-term sequelae in pediatric burn patients.

## Methods

### Study design and patient cohort

We conducted a retrospective study in patients under 18 years with acute burns admitted to our Pediatric Burn Unit between January 2015 and December 2021 who required surgical debridement of the burns. Exclusion criteria were the absence of clinical data or laboratory data at admission. Patients admitted to our unit for treatment of burn sequelae were also excluded. The study protocol was approved by the Institutional Review Board and Ethics Committee of our institution. Due to the retrospective nature of the study and the absence of human samples, it was not necessary to obtain informed consent, in accordance with institutional ethical guidelines.

### Data collection

We analyzed demographic variables (age, sex, weight), burn features (etiology, burned total body surface area [TBSA], burn depth and location), time elapsed from burn injury to initiation of treatment (surgical debridement) at our Burn Unit, acute treatment performed (surgical debridement and application of skin substitute) and chronic treatment (need for grafting or re-interventions). Laboratory data were derived from the blood tests conducted upon the patient’s arrival at the Emergency Department. These tests encompassed a complete blood count, including leukocyte count and absolute and relative values of neutrophils, lymphocytes, monocytes, basophils, and eosinophils. The NLR was obtained by calculating the proportion of the absolute value of neutrophil count and the absolute value of lymphocyte count. Additionally, biochemistry parameters such as ion levels, glucose, urea, and fibrinogen, along with C-reactive protein (CRP) levels, were assessed. Development of acute complications (need for reoperation, absence of epithelialisation) and chronic complications (pathological scarring, hypertrophy formation, keloids or retractions) were collected. Finally, the need for treatment of these complications and the type of treatment used (pressotherapy, corticosteroid injection, Z-plasty, grafts, etc.) were analysed. Patient medical records were meticulously examined to identify any short or long-term complications, as well as any surgical reintervention.

### Statistical analysis

Data were processed with Microsoft Excel version 2010 software (Redmond, WA, USA), and *analyzed* with SPSS Statistic version 23 (Chicago, IL, USA). To evaluate the distribution of numerical variables, the Shapiro-Wilk and Kolmogorov-Smirnoff tests were employed. Continuous variables that did not adhere to a normal distribution were described using medians and interquartile range (IQR), while those that followed a normal distribution were presented as means and standard deviation. For normally distributed continuous variables, Student’s t-test for independent samples was used, and to analyze non-normally distributed continuous data, Mann-Whitney test was used. Discrete variables were expressed as frequency and percentage, and were analyzed using the Chi-square test, or Fisher’s test when the former could not be applied. Odds ratios (OR) were calculated with 95% confidence intervals. All statistical calculations were two-tailed and statistical significance was established at a p value < 0.05. Sensitivity and specificity for the diagnosis of the different short- and medium-long term complications of the different clinical and laboratory parameters collected were determined by receiver operating characteristic (ROC) curves, which were compared using the DeLong method [12]. Optimal cut-off point values of maximum diagnostic accuracy for each analytical parameter were determined by the Youden index (sensitivity + specificity − 1) [13].

## Results

A total of 342 patients were included (198 males, 144 females), with a median age of 27 months (IQR 15–83 months), and a mean weight of 14.3 kg (SD 5.2). Based on ethnicity, most patients were Caucasian (149 patients, 43.6%), followed by Arab (86 patients, 25.1%), Latino (72 patients, 21.1%), and African (35 patients, 10.2%). Thermal burns were the most frequent (98.0%), with 78.5% due to scalding. Regarding burn depth, 72.2% of patients had deep dermal involvement, while in 27.8% of cases the involvement was limited to superficial dermis. The median burned TBSA 8% (IQR 4–10), while the median time elapsed from burn injury to initiation of treatment was 3 h (IQR 2–5 h). All patients included in the study underwent initial surgical debridement of the burns and coverage with skins substitutes. Regarding the type of skin substitute employed, Ez-Derm^®^ was the most frequently used (44.4%), followed by Biobrane^®^ (30.5%) and Suprathel^®^ (25.1%). Burn features are shown in Table 1.

**Table 1:** Burns features

Type of burn; *n* (%) • Thermal • Chemical • Electrical	335 (98.0) (1.2) 3 (10.8)
**Scald; n (%)**	263 (78.5)
**Burn dermal depth; n (%)** **• Superficial partial-thickness** **• Deep partial-thcikness**	95 (27.8) 247 (72.2)
**TBSA burned (%); median (**IQR**)**	8 (4–10)
**Time since burn (hours); median (**IQR**)**	3 (2–5)
**Type of skin substitute** • **Ez-Derm** • **Biobrane** • **Suprathel**	152 (44.4) 104 (30.5) 86 (25.1)

TBSA, total body surface area; IQR, interquartile range

**Fig. 1:**
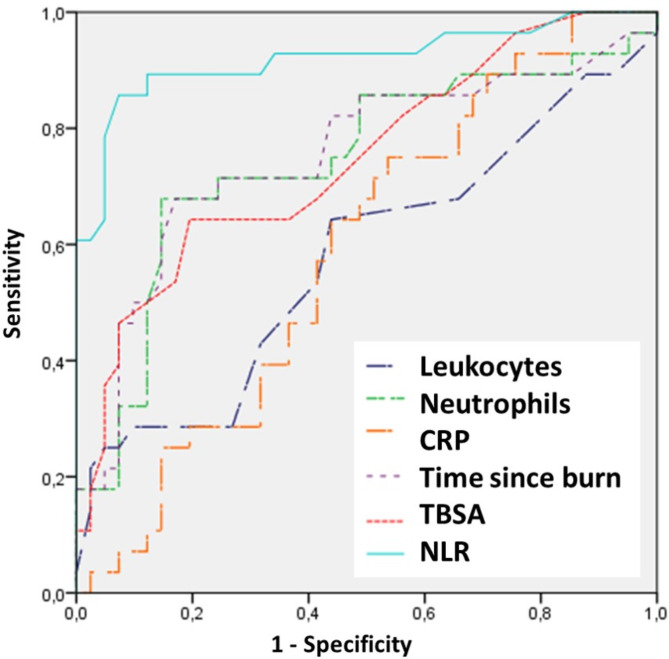
ROC curve for the need for escharectomy and skin graft

Concerning short-term outcome, 85 patients (24.9%) required escharectomy and grafting after the initial surgical debdridement. When analyzing predictors for the need for acute escharectomy and grafting using ROC curves, we found that NLR was the parameter with the highest AUC (0.920) and the highest sensitivity and specificity (90.0% and 88.4% respectively) when compared to other parameters such as burned TBSA, time to treatment since burn injury, leucocyte count, neutrophils or CRP, this difference being statistically significant (*p* < 0.001). Figure 1 shows the ROC curve for the need for escharectomy and grafting, while Table 2 shows the AUC, cut-off points and sensitivity and specificity values for the different parameters studied.

**Table 2:** Area under the curve (AUC), cut-off point, sensitivity and specificity of the different parameters to predict the need for escharectomy and grafting

	AUC	Cut-off point	Sensibility	Specificity	*P*-value
**NLR**	0.920	3.1	90.0	88.4	< 0.001
**Time since burn**	0.753	6.1	70.2	78.5	0.012
**Neutrophils**	0.746	11,293	70.2	78.5	0.024
**TBSA**	0.743	15	63.2	80.2	0.039
**CRP**	0.589	25,4	58.1	39.4	0.343
**Leukocytes**	0.577	15,140	55.4	38.7	0.639

NLR, neutrophil-to-lymphocyte ratio; TBSA, burned total body surface area; CRP, C-reactive protein

**Fig. 2:**
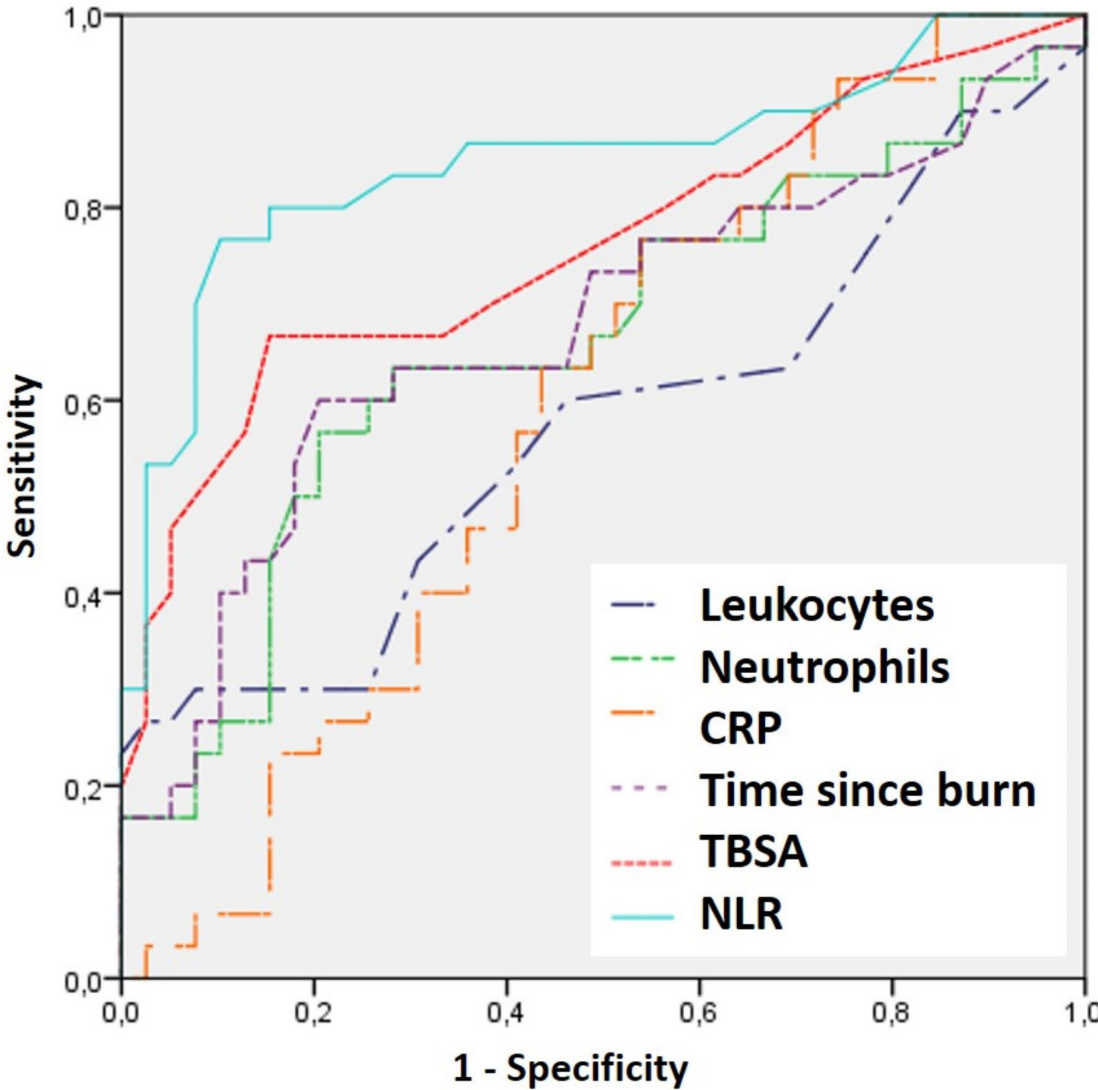
ROC curve for the development of long-term sequelae

Long-term sequelae were observed in 112 patients (32.7%) during a median follow-up of 4.5 years (IQR 2.7–6.8 years). The most frequent sequela was the presence of hypertrophy in 98 cases (28.7%), followed by the development of scar retraction in 21 cases (6.15%) and cicatricial alopecia in 15 cases (4.4%). In ROC curve analysis, we observed that NLR was the most sensitive and specific parameter presenting an AUC = 0.849 (80.4% sensitivity and 83.5% specificity), being significantly higher than the other parameters analyzed. Figure 2 shows the ROC curve for the development of long-term sequelae, and Table 3 shows the AUC, cut-off points and sensitivity and specificity values for the clinical and laboratory parameters studied.

**Table 3:** Area under the curve (AUC), cut-off point sensitivity and specificity of the different parameters to predict the development of long-term sequelae

	AUC	Cut-off point	Sensibility	Specificity	*P*-value
**NLR**	0.849	2.5	80.4	83.5	0.001
**TBSA**	0.759	9	68.3	81.3	0.025
**Neutrophils**	0.674	9,457	63.1	43.5	0.082
**Time since burn**	0.674	4.5	63.1	43.5	0.082
**CRP**	0.588	15.2	58.2	40.1	0.254
**Leukocytes**	0.570	14,458	57.1	38.8	0.348

NLR, neutrophil-to-lymphocyte ratio; TBSA, burned total body surface area; CRP, C-reactive protein

**Fig. 3:**
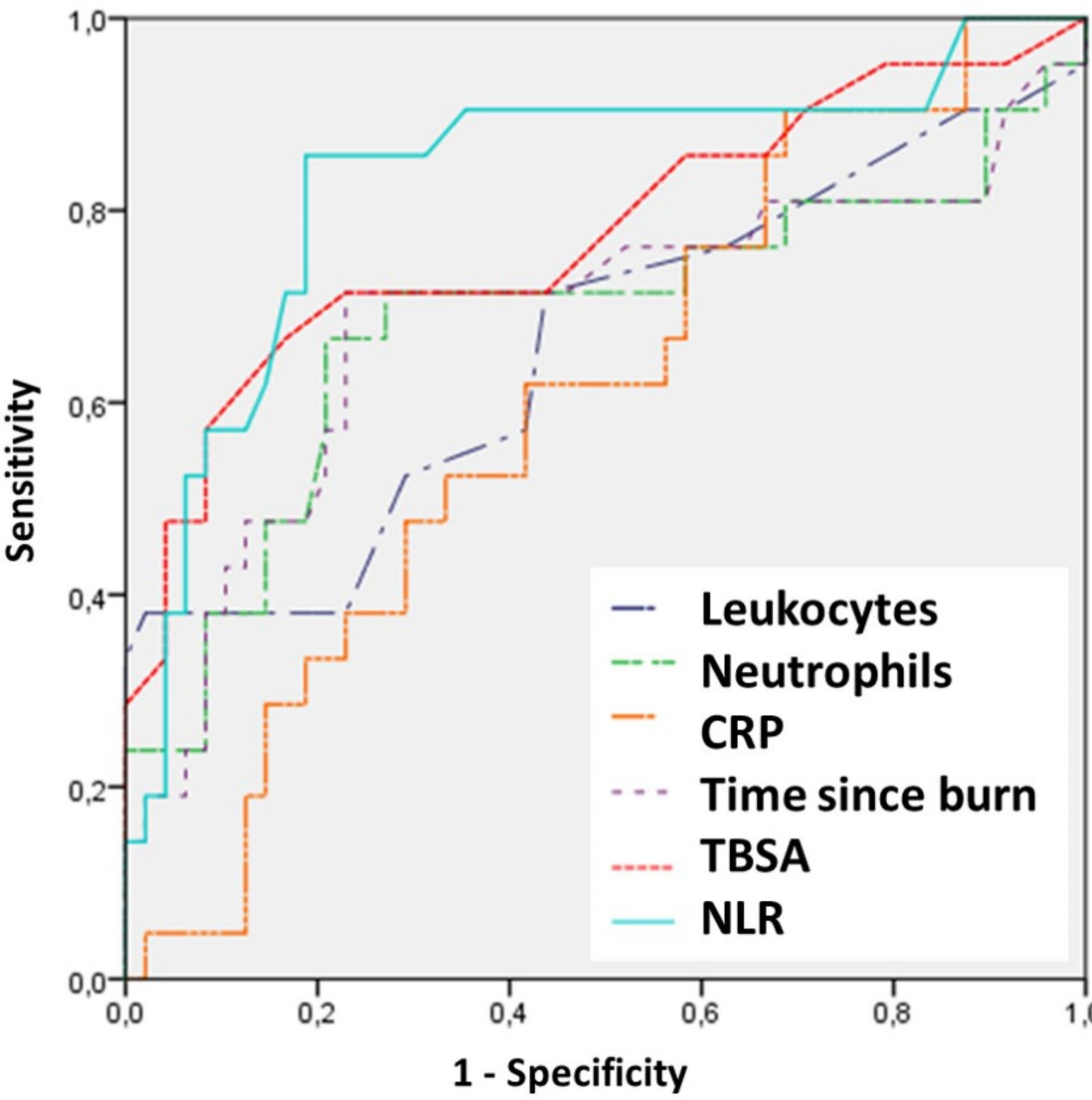
ROC curve for the need for surgery of the sequelae

Of the patients with long-term sequelae, 96 patients (28.1%) required some type of surgery during follow-up. Intralesional Triamcinolone injection in hypertrophic scars was the most frequent procedure in 67 patients (19.6%), followed by scar retraction release and skin graft in 18 cases (5.3%) and Z-plasties in 11 patients (3.2%). When analyzing the predictors of the need for surgery using ROC curves (Fig. 3), NLR again presented the highest sensitivity and specificity (83.5% and 80.9% respectively), with an AUC = 0.833, followed by TBSA (AUC = 0.777; sensitivity 73.1% and specificity 78.4%), which were significantly higher than for the other factors studied (Table 4).

**Table 4:** Comparison of the areas under the curve (AUC), cut-off point, sensitivity and specificity of different parameters to predict the need for long-term reintervention

	AUC	Cut-off point	Sensibility	Specificity	*P*-value
**NLR**	0.833	2.7	83.5	80.9	< 0.001
**TBSA**	0.753	11	73.1	78.4	< 0.001
**Time since burn**	0.688	5.4	71.4	77.9	0.079
**Neutrophils**	0.684	10,471	67.5	80.1	0.082
**Leukocytes**	0.659	14.240	64.3	59.4	0.194
**CRP**	0.602	50.2	62.4	58.1	0.287

NLR, neutrophil-to-lymphocyte ratio; TBSA, burned total body surface area; CRP, C-reactive protein

## Discussion

This study analyzes for the first time the role of the Neutrophil-to-Lymphocyte Ratio as a predictor of short- and long-term morbidity of childhood burns. It is the parameter with the highest sensitivity and specificity for predicting unfavorable evolution both in the short term (need for escharectomy and grafting) and in the long term (development of sequelae and need for surgical procedures), when compared with other clinical or laboratory data at admission.

Burns in pediatric patients are associated with different sequelae that can affect multiple areas such as physical, psychological, social, etc., of the patients and their families, presenting a challenge for physicians in terms of the difficulties of treatment and rehabilitation [1–3]. This reality is reflected in the sample of our study, where more than 30% of our patients presented long-term sequelae during follow-up and 28.1% required some type of surgical treatment of the sequelae. In an attempt to prevent or minimize short-term complications and long-term sequelae, it is necessary to search for predictors of unfavorable evolution, which are important for early identification of high-risk patients.

In this study we have evaluated clinical data, (such as burned TBSA and time elapsed between burn injury and initiation of treatment) [4, 14], as well as analytical data of inflammation (leukocytes, neutrophils, NLR, CRP.) to try to correlate their values with the risk of short-term complications (need for escharectomy and grafting), the development of long-term sequelae and the need for surgical treatment of the sequelae. A comprehension of the pathophysiology of burn injury is imperative to understand the mechanism underlying development of complications. Local injury triggers a cytokine cascade, that ultimately leads to a systemic inflammatory response which results in a hyperinflammatory state in the early stages of injury [15]. Elevated white blood cell counts, particularly with an increase in neutrophils, are commonly observed in the initial 24–48 h following burns and are usually proportional to the size of the burn injury. Platelet count, C-reactive protein (CRP), and procalcitonin levels in the blood have been highlighted as potential prognostic indicators for adult patients with severe burns [16, 17]. Recently, NLR has been identified as an important biomarker in several inflammatory diseases, showing a rapid increase within 6 h following acute physiological stress [18].

During the last years, the role of NLR in severe burns in adult patients has been analyzed, both in the prediction of sepsis and in the development of postoperative acute renal failure [10, 11]. The relationship between NLR and increased 90-day mortality in adults with 30% TBSA burns has also been described [19, 20]. However, to date, the role of NRL in the prediction of the development of short-term complications and long-term sequelae has not been evaluated, and to the best of our knowledge there are no studies in the pediatric population. Our study is, therefore, a novelty in this field, as it raises the possibility of using the NLR as a predictor of morbidity in burned children. In the immune system, the NLR level represents two compartments: that of neutrophils (innate immunity) and that of lymphocytes (adaptive immunity). Neutrophilia is caused by the rapid release of bone marrow reserves following the onset of inflammation. Additionally, lymphocyte levels decrease rapidly in the early stages of burn injury due to factors such as redistribution, increased attachment to vessel walls, and accelerated apoptosis [21]. NLR integrates information from both cell types by combining neutrophilia with lymphopenia, which enhances the predictive ability of this parameter compared to other inflammatory markers such as absolute values of leukocytes, neutrophils or CRP. In addition, NLR is easy to calculate using standard laboratory measurements of neutrophil and lymphocyte counts, which makes it easily integrable into clinical practice and cost- effective.

One of the strengths of our study is the very specific population included, namely burn patients under 18 years requiring initial surgical debridement and presenting clinical and laboratory data at admission, to correlate with the development of morbidity during postoperative and long-term follow-up. After a burn injury, painful triggers like burning agents or irritating substances in the burn area, activate pain receptors, and these signals travel through C and A delta nerve fibers to the spinal cord and reach the cerebral cortex, triggering the hypothalamic-pituitary-adrenal axis. This activation leads to higher levels of ACTH and cortisol, reducing lymphocyte counts and thereby decreasing immunity—especially in patients with severe or extensive burns. These effects can last through the entire healing process [22].

In this context, the need for escharectomy and grafting is the short-term outcome with the most significant associated comorbidity, since it entails a second surgical intervention after the initial debridement. The intensity of the initial acute inflammation, as reflected by the NLR, is correlated with the highest area under the curve (0.920), compared to the other parameters analyzed, with a sensitivity of 90% and a specificity of 88.4%, respectively. This may explain why patients with a higher NLR at admission have a higher risk of needing escharotomy and grafting. Along the same line, the development of long-term sequelae, such as hypertrophy, scar retraction or cicatricial alopecia are also correlated with the intensity of the initial acute inflammation at the time of burn injury. The extent of the local inflammatory response at the burn site triggers a proportional systemic inflammatory cascade, leading to an increase in inflammatory cells and cytokines at the injured site, and consequently to abnormal healing, mediated by the proliferation of fibroblasts and altered collagen fibers, leading to hypertrophy and tissue retraction at these areas. A similar correlation has recently been described in the evolution of primary vesicoureteral reflux (VUR) in children with acute pyelonephritis [23]. Patients with higher NLR at pyelonephritis diagnosis (and thus a more intense inflammatory response) are more likely to develop long-term sequelae, in a similar manner to what we have observed in burned children. The need for future surgical procedures in children with a history of burns is directly related to the development of long-term sequelae and their severity, since mild degrees of hypertrophy or scar contracture can be treated conservatively with gels or silicone sheets, whereas in more severe cases surgical procedures are necessary. This is a concern frequently raised by parents, which generates great anxiety. NLR was also the parameter that identified with the highest sensitivity and specificity those patients at high risk for the development of long-term sequelae and the need for surgical treatment during follow-up. Identifying beforehand which patients might be at a higher risk of developing severe long-term sequelae might help to initiate earlier and more aggressively preventive measures to avoid a potential unfavorable evolution in these selected cases.

Recent studies have highlighted the potential of the NLR as a predictor for prognostic outcomes in burn adult patients. In 2021, Qiu et al. and Setiawan et al. afterwards in 2022 revealed that an elevated NLR on the 3rd day post-burn was linked to a higher risk of mortality [24, 25]. More recently, Hung et al. determined that elevated NLR on the 7th day post-burn was significantly associated with an increased mortality risk [26]. In our series, all laboratory determinations were performed when the patient arrived at the Emergency Department, and we fortunately had no deaths, so it is not possible to analyze the role of inflammatory markers as predictors of mortality in our patients.

Limitations of this study are mostly due to its retrospective, single- center design. First, retrospective analysis allowed us to assess associations between clinical and laboratory data and postoperative outcomes, but this approach does not necessarily address causality. The retrospective nature also predisposes our conclusions to bias from unmeasured confounders. Second, there are limitations derived from the limited sample size, as the study was performed in a single institution. Our pediatric burn center treats approximately 500 pediatric burns annually, but there has been a decrease in the number of patients requiring hospital admission and surgical debridement since the COVID-19 pandemic, due to the implementation of new protocols that enable burn debridement under sedoanalgesia in the Emergency Department. These patients do not undergo laboratory tests, and therefore have not been included in the study. As this study was conducted at a single center, the treatment protocols for burns in the acute phase may differ from those at other burn centers. Therefore, these results need to be verified in further studies with larger samples from multiple centers. Finally, the absence of other studies in pediatric patients makes it difficult to compare our results with previous studies. Notwithstanding these limitations, this study represents the first attempt to examine and compare the predictive significance of systemic inflammatory indicators derived from blood cell counts in pediatric burn patients.

## Conclusion

NLR may be considered a useful predictor for the development of short-term complications and long-term sequelae in childhood burns. It may be useful in the prompt identification of high-risk patients to prevent adverse outcomes. Further multicenter studies with larger samples are needed to confirm these results.

## Data Availability

Data are available upon reasonable request to the authors.
